# UltraReporter for transforming spoken diagnostic cues into structured ultrasound reports with large language models

**DOI:** 10.1038/s41598-026-41439-w

**Published:** 2026-03-16

**Authors:** Pengyi Hao, Jilong Zhang, Siyuan Zhang, Fuli Wu, Shuyuan Tian

**Affiliations:** 1 College of Computer Science and Technology, College of Software, Zhejiang University of Technology, Hangzhou, 310014 China; 2 Department of Ultrasound, Tongde Hospital of Zhejiang Province, Hangzhou, 310012 China

**Keywords:** Health care, Mathematics and computing, Medical research

## Abstract

Ultrasound reporting remains a manual, time-consuming process prone to errors and variability. We present UltraReporter, a compact 8B-parameter LLM pipeline that converts real-time spoken cues into structured ultrasound reports. To overcome data scarcity, we developed a multi-agent framework for synthesizing high-quality Chinese cue-report pairs from unpaired narratives. The model was further refined through template-augmented fine-tuning and defect-oriented preference optimization to ensure institutional consistency and minimize hallucinations. Evaluated on 1,311 gold-standard cases, UltraReporter outperformed nine state-of-the-art LLMs, achieving superior clinical scores (e.g. accuracy: 4.82) and NLG metrics (BLEU-4: 89.42). In a blinded reader study, its reports surpassed chief physicians in quality across normal, common, and rare cases, and 72% of prospective reports were deemed equivalent to original ones. UltraReporter integrates seamlessly into clinical workflows, generating ready-to-use reports in 1 second, significantly reducing documentation burden and demonstrating strong potential for clinical integration.

## Introduction

Ultrasound has become the preferred imaging modality in many fields, such as obstetrics, cardiology, hepatobiliary, musculoskeletal and emergency nursing, because of its non-radiation, real-time, strong-portability and cost-effectiveness. Paradoxically, these same advantages have driven an explosive growth in examination volume, placing enormous pressure on ultrasonic physicians^1^. In current practice, a physician must simultaneously perform the scan and manually transcribe every measurement and impression into a structured report–a process that ranges from 30 seconds for a normal case to more than ten minutes for a complex case. Beyond being tedious and time-consuming, this step is prone to inter-operator variability and fatigue-induced errors, ultimately degrading data standardization and downstream secondary-use value.

Previous attempts to automate reporting have followed two main paradigms, neither of which aligns well with the clinical workflow. The first relies on automatic speech recognition of full dictated narratives, requiring physicians to speak entire paragraphs; this still consumes minutes per case and demands post-editing^2^. The second seeks image-to-report generation, but achieving “all-specialty” diagnostic is still beyond current capabilities^3^ and recent benchmarks still fall short of expert performance, risking hallucinated findings^4^.

In routine clinical practice, a physician calls out key findings (such as liver cyst, 1.2 by 1.1) while carrying out ultrasonic examination on the patient, and the assistant next to him captures these points in real time. At the end of the examination, the physician compiles the formal report. To seamlessly embed this workflow, we present UltraReporter that transforms the physician’s dictated cue into a fully structured, high-quality ultrasound report in less than one second. However, realizing this vision faces two key challenges. (1) Data scarcity and inconsistency: public corpora contain abundant narrative ultrasound reports, but no paired “spoken cue - structured ultrasound report” data; moreover, human-written reports vary widely in style, terminology and section granularity^5^. (2) Hallucination and clinical knowledge gaps: off-the-shelf LLMs lack domain-specific ontologies and may fabricate findings or omit crucial descriptions when only given brief cues^6,7^.

To address the two aforementioned challenges, we propose UltraReporter, an end-to-end LLM-driven pipeline that transforms concise, real-time spoken diagnostic cues into structured ultrasound reports. Unlike prior approaches, UltraReporter is designed for seamless integration into the clinical workflow, allowing physicians to maintain their natural examination rhythm.

To overcome the absence of paired “cue-report” data, we propose a multi-agent synthetic-data framework: a cue-simulator distills 30,000 de-identified narrative reports into concise physician-style clinical cues; a report-generator expands each cue into a full narrative; and a quality-evaluator, calibrated to board-certified standards, filters outputs based on clinical accuracy, completeness, and clarity, yielding 21,540 high-quality cue-report pairs. The resulting public corpus covers 324 anatomical sites and 3178 disease entities, serving as a foundational benchmark for the community.

The model is further refined through template-augmented fine-tuning, which grounds generation in institutional reporting standards, eliminating the phrase-book drift common in generic LLMs. Finally, we introduce Defect-Oriented Preference Optimization: the model critiques its own outputs, converts any measurement or reasoning error into a preference triplet, and re-trains via Direct Preference Optimization.

Across 1311 prospectively collected gold-standard examinations, UltraReporter surpasses nine state-of-the-art LLMs–including GPT-4.1, Claude-4-Sonnet and medical-tuned variants–by an average of 4.27% on LLM-based clinical rubrics and 53.9% on n-gram overlap metrics. In a blinded head-to-head preference study, three ultrasound specialists judged 84% of UltraReporter drafts to be equivalent or superior to original physician-authored reports, with 12% actively preferred for their completeness and adherence to institutional templates. The system is designed to be compatible with existing PACS and demonstrates the potential to generate reports with one-second latency–demonstrating that specialist-level documentation can be achieved without disrupting the scan, the clinician, or the clinical dollar.

Our contributions are threefold:

- We release the first open, 21k-pair Chinese “spoken cue $\rightarrow$ structured ultrasound report” corpus and a reproducible multi-agent pipeline that any institution can rerun to synthesize domain-specific training data without new annotations.
- We propose Template-Augmented SFT and Defect-Oriented Preference Optimization: two lightweight stages that implant institutional phrasing and self-correct measurement errors, lifting an 8B model above GPT-4.1 by 4.3% in accuracy with no extra hardware.
- We systematically validate UltraReporter’s superior performance and clinical utility. In a blinded study, 84% of UltraReporter drafts were judged equivalent or superior to physician reports, cutting documentation latency to in one second and demonstrating that real-time, AI-generated ultrasound reports are clinically acceptable and immediately deployable.

## Related work

### Medical report generation

In recent years, the field of Medical Report Generation (MRG) has witnessed rapid development, with the primary goal of alleviating the workload of radiologists and enhancing diagnostic efficiency. The majority of current research focuses on the “vision-language” multimodal framework, particularly on the generation of reports for chest X-ray images, such as Flamingo-CXR^8^, ViGPT2^9^, ATL-CA^10^, Teaser^11^, MA^12^, MCVGen^13^ and STREAM^14^. Regarding other imaging modalities, such as CT, MRI and ultrasound, research is relatively limited. BrainGPT^15^ is a clinical visual instruction tuning model. CT-ClearView^16^ shows a cross-modal alignment to address representation confusion for CT report generation. MVCL^17^, can enhance MRI report generation by utilizing symptom prior knowledge. For ultrasound generation, one work^3^ extracts knowledge from texts, which in turn guides visual feature alignment.

With the rapid development of large language models (LLMs), several studies have begun to leverage them to promote MRG. PromptMRG^18^ converts classification results into tagging prompts to explicitly guide the generation. Bootstrapping-LLMs^19^ narrows the gap between general models and the medical domain by providing a series of semantically associated reports for input X-ray images. LLM-RG4^20^ utilizes the flexible instruction-following ability and extensive common knowledge of LLMs to reduce irrelevant hallucination generation. MPO^21^ generates radiology reports that meet different preferences using multi-objective reinforcement learning. DADNET^22^ drafts preliminary reports using LLMs and then integrates them into a diffusion process to enhance report diversity. These efforts collectively propelled MRG from merely “generating correct sentences” to “generating clinically relevant, low-hallucination, and high-trust reports.”

However, existing literature on automated ultrasound report generation remains scarce. Compared with other imaging modalities, ultrasound relies more heavily on operator expertise, and images are noisier and artifact-laden, making fully automated interpretation unreliable. We therefore propose a clinically grounded alternative–generating structured reports directly from physician-dictated cues. To our knowledge, this paradigm has received virtually no prior attention and opens a new, clinically relevant avenue for medical report generation.

### Large language models in medical field

With the rapid development of general-purpose LLMs such as GPT-4^23^ and Llama3^24^, medical LLMs are typically developed through domain-specific pretraining on biomedical corpora followed by multitask instruction tuning, resulting in many notable models that analyze user inputs related to medical queries and generate professional explanations or provide relevant medical knowledge as responses, such as PubMedGPT^25^, Med-PaLM^26^, Med-PaLM2^27^, MedGemma^28^ and II-Medical^29^. For Chinese language medical LLMs, they were always trained on extensive Chinese medical corpora and integrated the Chinese medical knowledge graph to cultivate deep domain expertise, such as Baichuan-M1^30^, HuatuoGPT-o1^31^ and Zhongjing^32^.

LLMs are also being explored for diagnostic assistance, where they analyze symptom descriptions, test results, or medical images to generate potential diagnoses and support clinical decision-making. For example, the CHIEF^33^ focuses on tasks such as cancer cell detection, tumor origin identification, and prognostic prediction. BiomedGPT^34^, is a general biomedical vision-language foundation model. The MMedIns-Llama3^35^ shows exceptional performance in various clinical tasks. The MUSK^36^ shows potential in molecular biomarker prediction, melanoma recurrence prediction, and pan-cancer prognostic prediction. M3FM^37^, supports clinical diagnosis across disease reporting and classification tasks. With the development of multi-agent systems, recent advancements include collaborative diagnostic agents^38–40^ to process and analyze complex medical data, providing precise diagnostic suggestions.

Despite the proliferation of large language models in medical field, most lack an intrinsic grasp of department-specific reporting templates and stylistic conventions–particularly ultrasound. To address this, we give a compact, domain-expert model from state-of-the-art open-source LLMs, tailored exclusively to high-fidelity ultrasound report generation.

## Methods

**Fig. 1:**
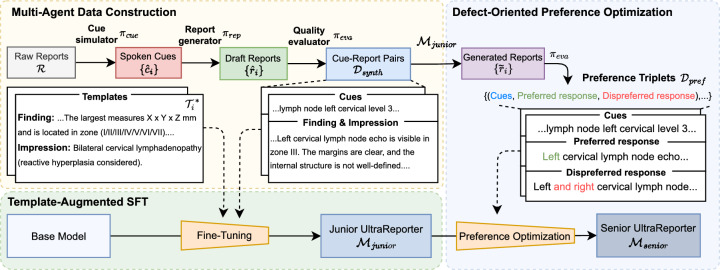
The training pipeline of UltraReporter. It proceeds in three stages: (1) multi-agent curation to synthesize a refined dataset, (2) template-enhanced SFT to produce the “Junior” model, and (3) defect-oriented preference optimization that elevates it to the “Senior” model by learning from its own outputs.

To achieve the end-to-end transformation from spoken diagnostic cues into structured ultrasound reports, we designed a training pipeline consisting of three core stages, as illustrated in Fig. 1. First, we employ a multi-agent collaborative framework to automatically synthesize a large-scale, high-quality dataset of “spoken cue-report” pairs, establishing the foundation for model training. Second, we utilize Template-Augmented Supervised Fine-Tuning (TA-SFT), integrating institutional standardized report templates into the training process. This enables the model (referred to as “Junior UltraReporter” at this stage) to master standard report formats and expressions. Finally, we introduce Defect-Oriented Preference Optimization (DOPO), which allows the model to learn from its own sub-optimal generated reports and self-correct. For clarity, we define the model derived from the supervised fine-tuning stage as “Junior UltraReporter,” and the final optimized model as “Senior UltraReporter.”

### Study design and protocol

To ensure methodological transparency and clinical rigor, we established a strict protocol for patient selection, data collection, and evaluation metrics. This study was conducted in accordance with the Declaration of Helsinki and was approved by the ethics committee of Tongde Hospital of Zhejiang Province.

Patient selection and criteria. The study focused on standard diagnostic reporting workflows for abdominal organs and superficial organs. These categories represent the majority of routine ultrasound examinations where structured reporting is most beneficial. Specialized examinations requiring distinct hemodynamic measurements or fetal biometry protocols–specifically cardiac ultrasound and obstetric ultrasound–were excluded from this study to maintain a unified reporting standard.

We employed a “Synthetic Validation / Real Test” strategy to address the scarcity of high-quality paired data while ensuring rigorous evaluation on real-world cases:

- Seed Dataset: A seed set of $N=377$ reports was purposively sampled by senior physicians to ensure coverage of diverse clinical scenarios and pathologies within the inclusion criteria. These cases served as the foundation for our multi-agent synthesis pipeline.
- Test Set: To evaluate performance in a realistic clinical setting, we collected a consecutive cohort of $N=1,311$ cases from daily clinical practice. This dataset reflects real-world disease prevalence and was strictly held out for testing.

Quantitatively, both datasets are heavily anchored in abdominal examinations: taking the test set as a representative example, the liver, gallbladder, spleen, and pancreas are documented in over 99% of reports, with kidneys appearing in approximately 80% of cases. Superficial organs (e.g. thyroid, about 50%) and genitourinary structures also feature prominently. This consistency ensures that our model is both seeded with and evaluated against data that accurately reflects the multi-organ complexity of daily diagnostic workflows.

Unlike traditional evaluations that rely on independent voting, we employed an “Expert Consensus Panel” to assess the clinical utility of the generated reports. Three ultrasound specialists reviewed each case. Instead of independent scoring, the experts discussed each case until a unanimous decision was reached regarding the comparative quality (Win/Tie/Lose) and the specific clinical scores. This consensus-based approach ensures that the ground truth for evaluation reflects a unified expert standard.

The primary endpoints included six clinical dimensions: Accuracy, Completeness, Relevance, Structure, Clarity, and Conciseness. Secondary endpoints focused on safety and error taxonomy. We explicitly categorized generation errors into “Omissions” (missing non-critical details) and Hallucinations. A confidence-based safety protocol was also analyzed to flag potential low-confidence generations for mandatory physician verification.

### Multi-agent data construction

The scarcity of high-quality paired real “spoken cue-structured report” data poses a fundamental challenge in medical report generation. Unlike traditional approaches that rely solely on manual annotation or simple data augmentation, we introduce a multi-agent collaborative framework that simulates the clinical review process to automatically generate “spoken cue - structured report” data at scale.

**Cue-simulator Agent (**$\pi _{cue}$**)**. To capture the diagnostic reasoning embedded in spoken cues, we compiled a seed set, $\mathscr {D}_{seed}$. Curated by three senior ultrasound physicians from Tongde Hospital of Zhejiang Province, each with over 10 years of experience. All data utilized in this study were approved by the ethics committee of Tongde Hospital of Zhejiang Province, in accordance with the Declaration of Helsinki. The requirement for informed consent was waived due to the retrospective nature of the study and the use of anonymized data. Drawing on their extensive clinical experience, they selected 377 representative ultrasound reports covering abdominal and superficial tissues. For these 377 reports, the three physicians dictated spoken-style diagnostic cues for each one, yielding a seed dataset of 377 (cue, report) pairs.

The Cue-simulator Agent is designed to mimic how physicians summarize key findings. It is an LLM (e.g. Qwen3-8B^41^) fine-tuned on $\mathscr {D}_{seed}$. Its task is to convert a large corpus of unannotated raw reports, $\mathscr {R}=\{u_i\}_{i=1}^{N}$, into concise diagnostic cues, $\{\hat{c}_i\}_{i=1}^N$, where $\hat{c}_i = \underset{c}{argmax}\ \ P_{\pi _{cue}}(c \mid u_i)$

This agent adheres to the following principles:

- The agent must identify and provide the core clinical conclusion and the essential observations that support it.
- The output cue should be brief, primarily composed of medical terms and key phrases. It should include critical measurements but omit units (e.g. “Liver cyst, 1.2 by 1.1”) to reflect spoken language patterns.

*Report-generator Agent (*$\pi _{rep}$**)** is an LLM fine-tuned on pairs ($\hat{c}_i$, $u_i$), where $\hat{c}_i$ denotes the cue synthesized by the Cue-simulator and $u_i$ denotes the original report. This training teaches the agent to expand a brief diagnostic cue into a complete, clinically coherent narrative.

During inference, the agent’s task is to expand a given cue $\hat{c}_i$ into a full, structured report $\hat{r}_i$. This generation process is represented as:

1 $$\begin{aligned} \hat{r}_i = \underset{r}{argmax}\ \ P_{\pi _{rep}}(r \mid \hat{c}_i). \end{aligned}$$

It operates under the following principle:

- The agent expands the cue into a structured report (containing finding and impression) that is strictly faithful to the provided information, without fabricating findings.

*Quality-evaluator Agent (*$\pi _{eva}$**)** is a large language model (e.g. Gemini-2.5-Pro^42^) tasked with guaranteeing the quality of synthetic data. It rates every generated report ($\hat{r}_i$) across six quality dimensions derived from the Physician Documentation Quality Instrument-9 (PDSQI-9)^43^. These dimensions were iteratively refined with board-certified ultrasound physicians using departmental ultrasound-reporting standards. They are defined as follows:

- *Accuracy:* The report must present findings that are both factually correct; any measurements, descriptors, or diagnostic statements must be free of clinical or numerical errors.
- *Completeness:* All clinically pertinent findings including lesion size, location, echogenicity, vascularity and adjacent-organ involvement are covered without omission.
- *Relevance:* Content is directly applicable to the clinical question and adds diagnostic value.
- *Structure:* The report is well-formed and structured in a way that helps the reader understand the patient’s clinical course.
- *Clarity:* Language is unambiguous, avoids unexplained abbreviations, and employs standardized descriptors so that referring clinicians, residents, and subspecialists can interpret the report without additional clarification.
- *Conciseness:* The report is succinct, avoiding redundancy while preserving all critical detail, supporting efficient reading in high-throughput environments.

The Quality-Evaluator Agent scores every candidate report $\hat{r}_i$ with a six-dimensional vector $s_i$. Each element in $s_i$ is corresponding to a 1-5 scale, where a higher value denotes better fulfillment of that dimension. Each dimension is assessed by comparing $\hat{r}_i$ with the original report $u_i$ and the spoken cue $\hat{c}_i$. The score vector is calculated as

2 $$\begin{aligned} s_i = \pi _{eva}(\hat{c}_i, u_i, \hat{r}_i). \end{aligned}$$

Any pair ($\hat{c}_i$, $\hat{r}_i$) whose score vector $s_i$ contains at least one element below the predefined threshold $\rho$ is discarded. This rigorous filtering process yields the final high-quality synthetic dataset, $\mathscr {D}_{synth}$. By eliminating low-scoring reports those marred by errors, omissions, or unclear phrasing, we ensure that the resulting dataset $\mathscr {D}_{synth}$ contains accurate, comprehensive content with clear, succinct expression, fully satisfying clinical reporting standards.

We constructed a high-quality “spoken cues–structured reports” dataset consisting of 21,540 pairs for the training and validation of UltraReporter. Each cue has an average of 31 Chinese characters, while the corresponding structured report averagely has 283 Chinese characters. The dataset covers 324 anatomical sites (e.g. liver, spleen), and 3,178 disease entities(e.g. Fatty liver, Gallbladder polyp). For the preference optimization stage, we further built 1,804 preference triplets(cue, high-quality report, low-quality report) derived from our Quality-Evaluator Agent. For the objective evaluation, we built an independent Gold Standard test set of 1311 pairs. This set, not derived from model-generated data, was curated by four experienced ultrasound physicians through multiple rounds of annotation and review to benchmark the model’s report generation performance. Table 1 summaries our dataset.

To explicitly validate our “Synthetic Validation / Real Test” experimental design, we analyzed the semantic distribution shift between the generated corpus and real-world clinical data. Given the scarcity of high-quality real-world samples, we reserved real data for testing to ensure rigorous evaluation, while using synthetic data for model validation.

**Fig. 2:**
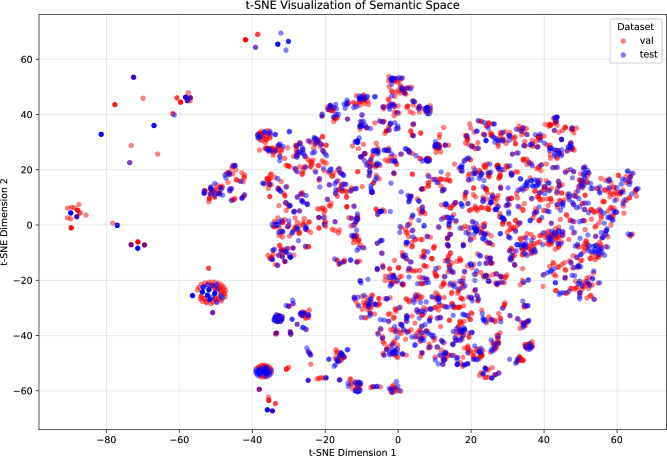
t-SNE visualization of the semantic feature space.

We performed a t-Distributed Stochastic Neighbor Embedding (t-SNE) analysis on the sentence embeddings of both the synthetic validation set and the held-out real-world test set. As illustrated in Fig. 2, the visualization reveals that the semantic features of the synthetic validation samples (red) effectively cover the feature space of the real-world test samples (blue). The significant overlap between the two distributions confirms that our multi-agent synthesized data successfully captures the linguistic diversity and clinical semantics of real-world scenarios, thereby serving as a valid proxy for model optimization and selection.

**Table 1:** Statistical summary of the dataset.

Metric	Value
Total cue-report pairs	21,540
Negative reports	3259 (15.13%)
Positive reports	18,281 (84.87%)
Avg. cue length	31
Avg. structured report length	283
Number of examination types	324
Number of disease entities	3178

### Training and preference optimization

#### Template-augmented supervised fine-tuning

While the synthetic dataset $\mathscr {D}_{synth}$ provides high-quality training pairs, clinical practice relies heavily on institutional templates to ensure report standardization and consistency. These structured templates encapsulate established diagnostic language and formatting conventions. To leverage this crucial source of domain knowledge and ensure our generated reports adhere to real clinical standards, we construct a template repository and design a corresponding retrieval strategy.

We maintain a repository $\mathscr {T}$ of 192 standard report templates collected from our collaborating institution. These templates cover a wide range of findings for both abdominal and superficial organ ultrasounds. Given a spoken cue $\hat{c}_i$, we retrieve the most relevant template(s) $\mathscr {T}_i^* \subset \mathscr {T}$. Since precise matching of pathology and anatomy is critical, we use a hybrid similarity score that prioritizes clinical correctness over pure semantic similarity.

For each cue $\hat{c}_i$ and template $t_j$, we first use a LLM(e.g. Gemini-2.5-Flash^42^) to extract anatomical sites and pathological findings, which are then mapped to sets of standard ICD-11 codes, denoted as ICD($\hat{c}_i$) and ICD($t_j$). The similarity is calculated as

3 $$\begin{aligned} \text {sim}(t_j, \hat{c}_i) = \alpha \cdot \mathbb {I}\left[ \frac{|ICD(t_j) \cap ICD(\hat{c}_i)|}{|ICD(t_j) \cup ICD(\hat{c}_i)|} > \tau \right] + (1-\alpha )\cdot \cos (\textbf{e}_{t_j}, \textbf{e}_{\hat{c}_i}), \end{aligned}$$

where $\mathbb {I}[\cdot ]$ is an indicator function that returns 1 if the pathological state of template $t_j$ matches the pathological state of cue $\hat{c}_i$ based on their ICD-11 codes, and 0 otherwise. $\textbf{e}_{t_j}$ and $\textbf{e}_{\hat{c}_i}$ are the embeddings of template $t_j$ and cue $\hat{c}_i$, respectively. $cos(\cdot )$ is the cosine similarity between $t_j$ and $\hat{c}_i$. According to this matching, we can get retrieved templates $\mathscr {T}^*_i = \{t^{(1)}_i, \dots , t^{(k)}_i\}$ for $\hat{c}_i$. Here $\alpha$ is a weighting hyper-parameter. We set $\alpha$ to 0.6, established through preliminary experiments to meet the clinical priority of precision over semantic proximity. This emphasis on an exact match is crucial for preventing serious diagnostic errors. Additionally, the retrieval threshold $\tau$ was set to 0.5. Given the weighting scheme, this threshold effectively filters out templates with mismatched pathological codes while ensuring sufficient semantic relevance for the remaining candidates.

The Junior UltraReporter $\mathscr {M}_{junior}$ is trained on Qwen3-8B by minimizing the standard negative log-likelihood loss of generating the ground-truth report $\hat{r}_i$, conditioned on the spoken cue $\hat{c}_i$ and the retrieved templates $\mathscr {T}^*_i$:

4 $$\begin{aligned} \mathscr {L}_{SFT} = - \sum log \ P(\hat{r}_i \mid \hat{c}_i, \mathscr {T}^*_i; \Theta ), \end{aligned}$$

where $\Theta$ represents the parameters of the model.

#### Defect-oriented preference optimization

Although $\mathscr {M}_{junior}$ can generate ultrasound reports from concise cues, it may still struggle with the implicit reasoning required in clinical practice. For instance, in a cue like “gallbladder enlarged..., gallstones. size 8.6 x 4.6...”, the subject for the measurement “size 8.6 x 4.6” is omitted. An experienced physician immediately infers from the context and the magnitude of the values that 8.6 x 4.6 refers to the gallbladder, not the gallstones. However, the SFT-only model might incorrectly associate this measurement with the gallstones, leading to a significant diagnostic misinterpretation.

To address these subtle yet critical failures in reasoning, we introduce a preference optimization stage. The model learns by identifying its own defects and correcting them. First, we use the trained Junior UltraReporter, $\mathscr {M}_{junior}$, to generate a new set of reports $\{\tilde{r}_i\}$ for all cues $\hat{c}_i$ in our dataset $\mathscr {D}_{synth}$.The Quality-Evaluator Agent then re-evaluates these newly generated reports, focusing on the clinically critical dimensions of “Accuracy” and “Completeness.” If a report $\tilde{r}_i$ scores below a strict threshold $\rho$ on either dimension, it is identified as a suboptimal response.

Based on this mechanism, we construct a preference triplet ($\hat{c}_k$,$u_k$,$\tilde{r}_k$), where the ground-truth report $u_k$ is the preferred response, and the model-generated suboptimal report $\tilde{r}_k$ is the dispreferred response. This process yields a preference dataset, $\mathscr {D}_{pref}=\{(\hat{c}_k,u_k,\tilde{r}_k)\}$, consisting of 1,804 triplets. Unlike the SFT data, this set is naturally enriched with rare pathologies and ambiguous common cases, as $\mathscr {M}_{junior}$ is already proficient at handling straightforward scenarios. This balanced distribution explicitly teaches the model to distinguish between high-quality and subtly flawed reports. We then fine-tune $\mathscr {M}_{junior}$ on $\mathscr {D}_{pref}$ using the Direct Preference Optimization (DPO) loss function. It directly optimizes the model to increase the likelihood of preferred responses while decreasing the likelihood of dispreferred responses. The loss is defined as:

5 $$\begin{aligned} \mathscr {L}_{DPO}=-\mathbb {E}_{(\hat{c}_k,u_k,\tilde{r}_k)\sim \mathscr {D}_{pref}}[\log \sigma (\beta \log \frac{\pi _\theta (u_k|\hat{c}_k)}{\pi _{ref}(u_k|\hat{c}_k)} -\beta \log \frac{\pi _\theta (\tilde{r}_k|\hat{c}_k)}{\pi _{ref}(\tilde{r}_k|\hat{c}_k)})] \end{aligned}$$

where $\pi _\theta$ is the policy model being optimized (initialized from $\mathscr {M}_{junior}$), $\pi _{ref}$ is a frozen reference copy of $\mathscr {M}_{junior}$ to prevent model drift, $\beta$ is a temperature parameter, and $\sigma$ is the sigmoid function. This final stage produces the Senior UltraReporter, $\mathscr {M}_{senior}$.

### End-to-end clinical workflow

**Fig. 3:**
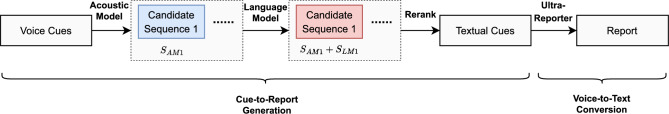
The end-to-end clinical workflow.

The preceding sections have detailed the multi-stage process for training and optimizing the UltraReporter model. This section describes the proposed end-to-end operational workflow for the future clinical deployment of $\mathscr {M}_{senior}$. While our current experimental validation operates as a standalone prototype, this workflow illustrates how the system is designed to seamlessly integrate. The workflow is designed to seamlessly integrate into the existing ultrasound examination process, transforming a physician’s spoken observations into a structured report ready for final validation. As shown in Fig. 3, the process unfolds in two key stages:

1. *Voice-to-text conversion* This stage is engineered to address the significant challenges inherent in clinical speech recognition, such as ambient noise from ultrasound equipment, variations in physician accents and diction, and the prevalence of highly specialized medical terminology. The captured audio is processed by a robust speech recognition pipeline designed to overcome these obstacles and ensure high transcriptional accuracy.

The pipeline operates in two phases. First, an Acoustic Model, trained to be resilient to background noise, analyzes the raw audio signal. It generates multiple candidate transcription sequences, each representing a phonetically plausible interpretation of the speech. Each candidate is assigned an acoustic model score $S_{AM}$, which represents the log-likelihood of the observed acoustic features X given a candidate word sequence W:

6 $$\begin{aligned} S_{AM} = \log P(X|W). \end{aligned}$$

This score reflects the fidelity of the transcription to the original audio signal.

However, acoustic information alone is often insufficient to disambiguate medical jargon from phonetically similar common words (e.g. distinguishing “portal vein” from “portal pain”). Therefore, the candidates are then evaluated by a domain-specific Probabilistic Language Model. This model is heavily fine-tuned on a large corpus of clinical texts, giving it a deep understanding of the syntax and semantics of ultrasound diagnostics. It assigns a language model score $S_{LM}$, representing the prior log-probability of the word sequence $W=(w_1, w_2,..., w_L)$ itself. This is typically calculated using an n-gram approximation:

7 $$\begin{aligned} S_{LM} = \frac{1}{L} \sum _{i=1}^{L} \log P(w_i | w_{1}, ..., w_{i-1}). \end{aligned}$$

This score assesses the clinical and linguistic plausibility of the candidate, effectively prioritizing medically coherent phrases while filtering out filler words or contextual errors.

To derive the final transcription, the system integrates both acoustic and linguistic evidence through a weighted reranking mechanism. The total score for each sequence is calculated as:

8 $$\begin{aligned} {tot}_s = \psi \cdot S_{LM} + (1-\psi ) \cdot S_{AM}. \end{aligned}$$

By assigning a significant weight $\psi$ to the domain-specific language model, the system resolves ambiguities and selects the sequence that is not only acoustically accurate but also clinically meaningful. The sequence with the highest total score is then selected as the definitive text-based cue for the next stage.

2. *Cue-to-report generation* The resulting high-fidelity text-based cue is then fed directly as input to the fully optimized UltraReporter model $\mathscr {M}_{senior}$. Leveraging its extensive training on the synthesized corpus and preference data, the model expands the concise cue into a complete, structured clinical report, populating the standard “finding” and “impression” sections. This workflow significantly reduces the manual reporting burden while maintaining full clinical oversight.

### Confidence-based safety and human-in-the-loop verification

To mitigate the opacity inherent in end-to-end neural generation and prevent automation bias, UltraReporter is strictly designed as a Human-in-the-Loop assistant rather than an autonomous closed-loop system. We implemented a confidence-based safety protocol that leverages the model’s intrinsic uncertainty quantification to ensure clinical safety.

Specifically, for each generated token $w_i$ in the structured report $\hat{r}$, we compute a confidence score $S_{conf}$ ($w_t$) based on the probability distribution from the model’s final softmax layer:

9 $$\begin{aligned} S_{conf}(w_t) = P(w_t | w_{<t} , \hat{c}, \theta ). \end{aligned}$$

We establish a strict safety threshold $\tau _{safe}$. During the inference phase, the system automatically monitors the generation of clinically critical entities, including numerical measurements, anatomical locations, and pathological descriptors. Any segment generated with a confidence score $S_{conf}(w_t) < \tau _{safe}$ is immediately flagged as uncertain.

In the physician’s user interface, these low-confidence segments are rendered with distinct visual highlighting (e.g. color-coded warnings), as illustrated in Supplementary Fig. 1. This mechanism proactively directs the physician’s attention to potential ambiguities or hallucinations, enforcing mandatory human verification for uncertain content. Consequently, the workflow ensures that while the drafting process is automated, the final clinical validation remains transparent, traceable, and under the full control of the expert sonographer.

### Experimental setup

To strictly evaluate the algorithmic performance and inference latency, experiments were conducted in a standalone environment, isolating the model from the hospital network infrastructure. All experiments were conducted on a high-performance server equipped with two Intel Xeon Gold 6530 CPUs, four NVIDIA L40 GPUs, and 256 GB of RAM. The system runs on Ubuntu 20.04, and the experiments were implemented based on the LlamaFactory^44^ v0.9.3 framework. The threshold for the Quality-Evaluator Agent is set to $\rho$ = 3. Supervised fine-tuning phase used the AdamW^45^ optimizer with a learning rate of 1e-4, a batch size of 16, and a LoRA rank of 32. Preference optimization employed a learning rate of 1e-5, a batch size of 8 and temperature parameter $\beta$ = 0.1. In the speech-to-cue conversion pipeline, the weight of the domain-specific language model $\psi$ is set to 0.5. To balance evaluation robustness with computational efficiency, we conducted five independent training runs of the same model using five different random seeds. We chose the model that achieved the best performance on the validation set and report its evaluation results on the test set. The acoustic model is implemented using a pre-trained WeNet [Cite WeNet]. The language model is an n-gram model trained on AISHELL-1^46^, Huatuo Encyclopedia QA^47^, and HuatuoGPT SFT Data^48^ to enhance domain adaptation.

To ensure a fair and comprehensive comparison, we evaluated nine state-of-the-art benchmark LLMs including medical (HuaTuoGPT-o1-7B^31^, MedGemma-4B^28^, II-Medical-8B^29^), general (Qwen3-14B^41^, Gemini-2.5-Flash^42^, DeepSeek-V3^49^, Mistral-small-24B^50^), and commercial (Claude-Sonnet-4^51^, GPT-4.1^52^). The performance of each model was assessed using two complementary metrics: (1) Clinical dimensions based on LLM(GPT-4.1), every generated report is scored on a 1-5 scale across Accuracy (Acc.), Completeness (Com.), Relevance (Rel.), Structure (Str.), Clarity (Cla.), and Conciseness (Con.); and (2) NLG metrics, ROUGE-L and BLEU-1/2/3/4 are computed against reference reports. BLEU-1/2/3/4 and Rouge-L are abbreviated as B-1, B-2, B-3, B-4 and R-L in the later subsections. All baseline models were evaluated using identical inference parameters as UltraReporter. Furthermore, the input prompts for all baselines incorporated the same institutional templates used by UltraReporter, ensuring that all models had access to equivalent contextual information.

## Results

**Table 2:** Comparison with state-of-the-art general, medical, and commercial models.

Model	LLM evaluation						NLG metric				
	Acc.	Com.	Rel.	Str.	Cla.	Con.	B-1	B-2	B-3	B-4	R-L
Qwen3-14B	4.36	4.33	4.62	4.83	4.80	3.90	39.04	25.64	17.22	11.49	28.32
Mistral-small-3.2-24B	3.98	3.88	4.41	4.77	4.53	3.33	36.84	24.09	16.04	10.62	29.20
Gemini-2.5-Flash	4.27	4.41	4.65	4.94	4.88	3.87	37.28	26.16	18.52	13.02	30.99
DeepSeek-V3-671B	4.56	4.56	4.93	4.98	4.96	3.97	43.00	29.82	21.30	15.25	38.29
Claude-Sonnet-4	4.50	4.15	4.79	4.96	4.88	3.93	55.91	41.07	29.64	20.81	44.13
GPT-4.1	4.66	4.20	4.85	4.96	4.93	3.91	49.49	34.35	24.07	16.64	36.70
HuaTuoGPT-o1-7B	3.52	2.25	3.47	3.60	3.88	2.95	42.59	26.94	17.73	11.72	27.19
MedGemma-4B	3.55	2.01	2.81	3.28	2.98	2.38	29.03	19.29	13.21	9.24	29.47
II-Medical-8B	3.90	3.67	4.31	4.72	4.51	3.52	36.30	22.88	15.13	9.84	29.36
UltraReporter	4.82	4.82	4.97	5.00	4.99	4.18	94.46	92.55	90.89	89.42	93.76

**Table 3:** Detailed statistical comparison of UltraReporter against other models across six dimensions.

Dimension	Comparison model	Mean diff. ($\Delta$**)**	99.72% CI	t-test p-value	Wilcoxon p-value
Accuracy	vs. GPT-4.1	0.159	[0.116, 0.201]	< 0.001	< 0.001
	vs. Claude-Sonnet-4	0.328	[0.275, 0.377]	< 0.001	< 0.001
	vs. DeepSeek-V3	0.259	[0.218, 0.303]	< 0.001	< 0.001
Completeness	vs. GPT-4.1	0.621	[0.572, 0.670]	< 0.001	< 0.001
	vs. Claude-Sonnet-4	0.671	[0.623, 0.722]	< 0.001	< 0.001
	vs. DeepSeek-V3	0.261	[0.217, 0.304]	< 0.001	< 0.001
Relevance	vs. GPT-4.1	0.125	[0.100, 0.149]	< 0.001	< 0.001
	vs. Claude-Sonnet-4	0.183	[0.155, 0.211]	< 0.001	< 0.001
	vs. DeepSeek-V3	0.045	[0.023, 0.067]	< 0.001	< 0.001
Structure	vs. GPT-4.1	0.033	[0.022, 0.046]	< 0.001	< 0.001
	vs. Claude-Sonnet-4	0.036	[0.024, 0.048]	< 0.001	< 0.001
	vs. DeepSeek-V3	0.016	[0.002, 0.032]	0.003	0.003
Clarity	vs. GPT-4.1	0.056	[0.040, 0.074]	< 0.001	< 0.001
	vs. Claude-Sonnet-4	0.106	[0.086, 0.128]	< 0.001	< 0.001
	vs. DeepSeek-V3	0.025	[0.007, 0.045]	< 0.001	< 0.001
Conciseness	vs. GPT-4.1	0.266	[0.234, 0.298]	< 0.001	< 0.001
	vs. Claude-Sonnet-4	0.248	[0.216, 0.278]	< 0.001	< 0.001
	vs. DeepSeek-V3	0.210	[0.183, 0.237]	< 0.001	< 0.001

**Fig. 4:**
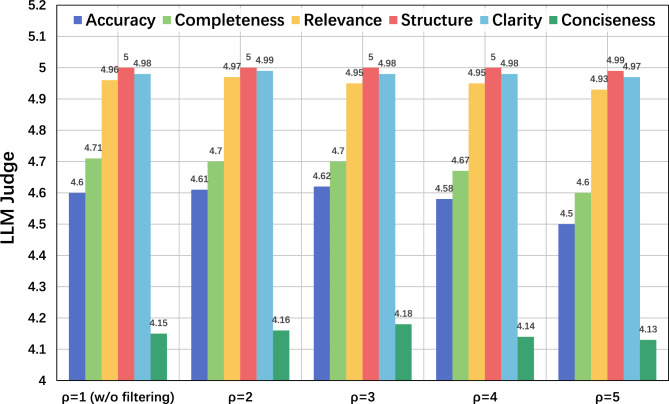
Impact of the quality filtering threshold ($\rho$) on model performance. The x-axis represents the threshold score used by the Quality-Evaluator Agent to filter synthetic data; $\rho = 1$ (w/o filtering) indicates the baseline with no data filtering, while higher values indicate stricter quality control. The y-axis displays the Clinical Quality Score evaluated by GPT-4.1 on a Likert scale of 1 to 5 (where 5 indicates expert-level quality). The results demonstrate that $\rho =3$ offers the optimal balance between data quality and quantity, as performance significantly degrades with overly strict thresholds ($\rho > 4$).

**Fig. 5:**
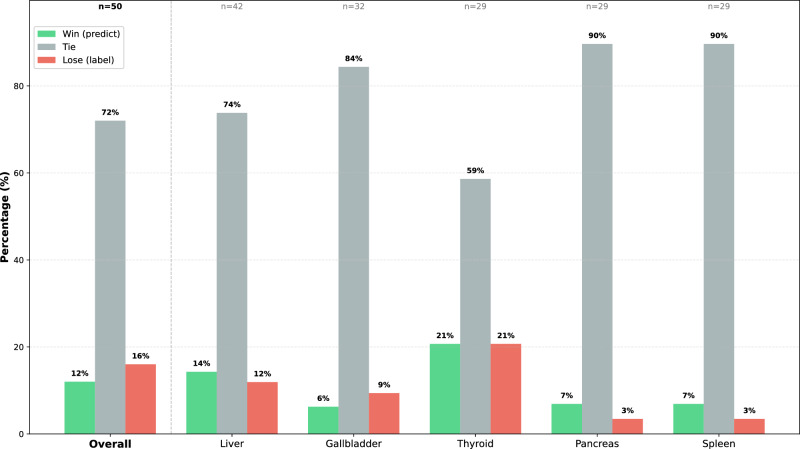
Clinician preference evaluation results.

**Table 4:** Performance of different base models after fine-tuning on $\mathscr {D}_{synth}$.

Model	State	LLM evaluation						NLG metric				
		Acc.	Com.	Rel.	Str.	Cla.	Con.	B-1	B-2	B-3	B-4	R-L
Gemma3-4B	Base	3.38	2.45	3.32	3.84	3.66	2.64	27.76	17.05	10.98	7.08	22.27
	Fine-tune	4.55	4.69	4.86	5.00	4.96	4.09	91.50	88.78	86.44	84.40	90.31
MedGemma-4B	Base	3.55	2.01	2.81	3.28	2.98	2.38	29.03	19.29	13.21	9.24	29.47
	Fine-tune	4.39	4.63	4.84	4.99	4.94	3.96	91.85	89.17	86.87	84.87	90.56
Llama3.1-8B	Base	2.95	2.14	2.94	3.74	3.39	2.51	32.55	20.39	13.29	8.79	23.64
	Fine-tune	4.46	4.70	4.87	4.99	4.95	3.98	91.91	89.11	86.68	84.55	90.20
Qwen3-8B	Base	3.85	3.87	4.42	4.75	4.61	3.39	40.54	28.20	19.55	13.29	32.91
	Fine-tune	4.56	4.70	4.87	4.98	4.90	4.02	92.99	90.50	88.35	86.48	91.93

“Base” indicates zero-shot performance with templates; “Fine-tune” shows post-training results.

**Table 5:** Ablation study of training stages in UltraReporter.

Stages	LLM evaluation						NLG metric				
	Acc.	Com.	Rel.	Str.	Cla.	Con.	B-1	B-2	B-3	B-4	R-L
SFT only	4.56	4.70	4.87	4.98	4.90	4.02	92.99	90.50	88.35	86.48	91.93
TA-SFT only	4.62	4.78	4.95	5.00	4.98	4.18	93.71	91.72	89.99	88.47	93.38
SFT + DOPO	4.74	4.83	4.95	5.00	4.97	4.10	93.46	91.32	89.49	87.87	93.08
TA-SFT + DOPO	4.82	4.82	4.97	5.00	4.99	4.18	94.46	92.55	90.89	89.42	93.76

### Main results

The performance of UltraReporter was evaluated against nine benchmark LLMs. As presented in Table 2, UltraReporter achieved the highest scores across all evaluated metrics. In the clinical evaluation, it scored 4.82 in Accuracy, 4.97 in Completeness, and 5.00 in Relevance. For Natural Language Generation (NLG) metrics, UltraReporter achieved a BLEU-4 score of 89.42 and a ROUGE-L score of 93.76.

To confirm the reliability of these findings, statistical significance tests were performed. As shown in Table 3, single-sample t-tests and Wilcoxon signed-rank tests revealed that UltraReporter’s performance advantage over leading models like GPT-4.1 and Claude-Sonnet-4 was statistically significant across all six criteria (p < 0.001). Specifically, we applied the Holm-Bonferroni correction to the p-values derived from both single-sample t-tests and Wilcoxon signed-rank tests. Additionally, bootstrap confidence intervals were adjusted using the Bonferroni correction to ensure robust statistical inference. The resulting 99.72% confidence intervals for the mean score differences all lay entirely above zero, indicating that the observed gains are systematic and not due to sampling noise.

Experiments were also conducted to measure the impact of fine-tuning on the specialized corpus. Table 4 shows that after fine-tuning, all models exhibited substantial improvements across all metrics. For instance, accuracy scores rose by 3.2-26% and completeness by 5.2-56.2%. The fine-tuned models’ BLEU-4 scores converged to a narrow range between 84.40 and 86.48.

### Ablation and hyperparameter analysis

**Fig. 6:**
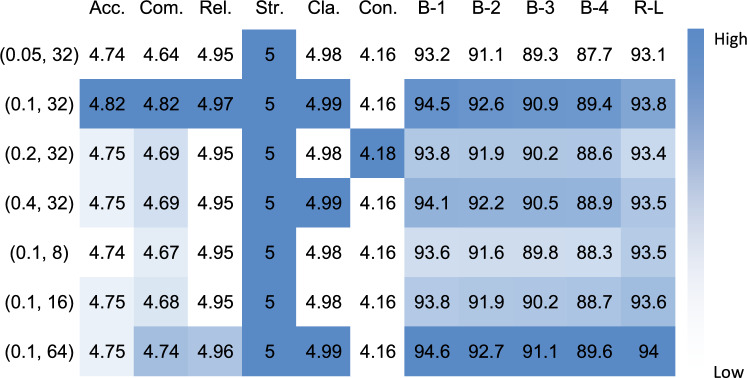
Hyperparameter sensitivity analysis for the UltraReporter model. The heatmap illustrates model performance under different hyperparameter configurations. (Left Y-axis): The configurations are denoted as tuples (DPO Temperature $\beta$, LoRA Rank) used during the training phase. (Top X-axis): Columns represent Clinical Dimensions (scored 1–5): Acc. (Accuracy), Com. (Completeness), Rel. (Relevance), Str. (Structure), Cla. (Clarity), and Con. (Conciseness); and NLG Metrics (scored 0–100): B-1/2/3/4 (BLEU-1/2/3/4) and R-L (ROUGE-L). (Color Scale): Cell shading indicates relative performance within each metric column, where darker blue signifies higher (better) scores. The analysis shows the model is robust to hyperparameter variations, with the (0.1, 32) configuration achieving optimal stability.

Ablation experiments were conducted to validate the contributions of UltraReporter’s core components: Template-Augmented Supervised Fine-Tuning (TA-SFT) and Defect-Oriented Preference Optimization (DOPO). The results are quantified in Table 5. The full model “TA-SFT + DOPO”) achieved the highest scores, with an Accuracy of 4.82 and a BLEU-4 of 89.42. In comparison, the “SFT only” model achieved an Accuracy of 4.56 and a BLEU-4 of 86.48.

The analysis was extended to the hyperparameter $\rho$, which controls the quality filter in the data construction pipeline. As illustrated in Fig. 4, model performance remained stable as $\rho$ increased from 1 (no filtering) to 3. However, performance, particularly in Accuracy and Completeness, declined sharply with $\rho > 3$. Consequently, $\rho = 3$ was established as the optimal balance between data quality control and data sufficiency. The sensitivity to other critical hyperparameters, LoRA rank and DPO temperature ($\beta$), was also explored, showing that the model demonstrated notable robustness with small performance variations under all tested configurations.

### Human-model comparison

**Table 6:** Report quality comparison between physicians and models, stratified by case type: normal(top), common(middle), and rare(bottom).

Reporter	Acc.	Com.	Rel.	Str.	Cla.	Con.
Resident	4.40	4.30	4.65	5.00	4.85	3.90
Attending	4.80	4.65	4.80	5.00	4.85	3.80
Chief	4.85	4.75	5.00	5.00	5.00	3.95
Claude	4.75	4.55	4.90	5.00	5.00	4.00
Deepseek	4.70	4.60	4.95	5.00	5.00	4.00
UltraReporter	4.95	4.80	5.00	5.00	5.00	4.45
Resident	3.95	3.85	4.45	4.90	4.55	3.50
Attending	4.25	4.15	4.60	5.00	4.65	3.65
Chief	4.25	4.15	4.70	4.95	4.85	3.80
Claude	4.45	3.75	4.80	4.95	4.95	4.00
Deepseek	4.55	4.40	4.90	5.00	5.00	4.00
UltraReporter	4.80	4.50	4.90	5.00	5.00	4.05
Resident	3.50	3.38	4.12	5.00	4.50	3.50
Attending	3.88	3.50	4.38	5.00	4.50	3.38
Chief	4.00	4.25	4.88	5.00	5.00	4.00
Claude	4.38	3.25	4.25	5.00	4.75	3.88
Deepseek	4.38	3.62	4.62	4.88	4.88	3.50
UltraReporter	4.63	4.50	4.88	5.00	4.88	4.13

A blind reader study was conducted to compare reports generated by physicians and models across 144 cases of varying difficulty (normal, common, rare). Reports were generated by nine ultrasound physicians (residents, attendings, chiefs) and three models. All reports were then evaluated across six clinical dimensions. The results are presented in Table 6.

- Normal Cases: All models and physician groups produced high-quality reports. UltraReporter achieved the highest scores in this category on Accuracy (4.95), Completeness (4.80), and Conciseness (4.45).
- Common Cases: UltraReporter’s scores (e.g. 4.80 Accuracy) were higher than those of all physician groups, including chief physicians (4.25 Accuracy).
- Rare Cases: UltraReporter again scored higher across all six metrics compared to both chief physicians and the other models. For example, UltraReporter’s Accuracy was 4.63, while the chief physicians’ was 4.00.

### Clinician preference evaluation

To further evaluate the clinical utility of Ultra Reporter in a real-world setting, five sonographers from Zhejiang Provincial Tongde Hospital were invited to use the system in their daily practice. While performing examinations, the physicians verbally described the key findings from the ultrasound images. Our system processed this speech stream to extract critical information, enabling UltraReporter to generate a comprehensive draft of the ultrasound report immediately upon completion of the scan. Our evaluation dataset consisted of 50 report pairs, each comprising a randomly selected Ultra Reporter-generated report and the corresponding original report authored by an ultrasonographer for the same patient. To facilitate an impartial assessment, the two reports within each pair were anonymized and randomly assigned identifiers (“A” or “B”). Three expert radiologists specializing in ultrasound then performed a blinded comparison of all 50 pairs, rendering a judgment on which report exhibited superior quality. Without knowing the origin of either report, the physicians were asked to choose which of the two reports was of higher quality (“Win” for UltraReporter or “Lose”), or to rate them as being of comparable quality (“Tie”). As shown in Fig. 5, the overall results from 50 comparisons demonstrate that UltraReporter’s generated reports are highly competitive with those written by physicians. In 12% of the cases, physicians preferred the generated report (“Win”), while in 16% of cases, they favored the original report (“Lose”). Notably, in the vast majority of cases–72%–the physicians judged the two reports to be of equivalent quality (“Tie”), indicating that UltraReporter consistently performs at a level comparable to human experts.

A more granular analysis at the organ level reveals a similar trend. For instance, in the evaluation of liver reports (n=42), UltraReporter’s win rate (14.3%) surpassed its loss rate (11.9%). For reports concerning the pancreas (n=29) and spleen (n=29), the tie rate reached nearly 90%, underscoring the high degree of reliability and consistency of the generated reports for these organs.

## Discussion

This study developed and validated UltraReporter, an LLM-driven pipeline that transforms spoken diagnostic cues into structured clinical reports. The results demonstrate that UltraReporter’s performance is superior to other leading general and medical LLMs on this specialized task.

The data in Table 2 shows that UltraReporter overwhelmingly outperforms other models, a finding that is not only numerical but also statistically significant ($p<0.001$ against major competitors, as detailed in Table 3). This high degree of statistical reliability, confirmed through significance testing and confidence interval analysis, underscores the robustness of the model’s advantage. This superior performance, achieved with a relatively small 8B parameter model, can be attributed to the specialized, multi-stage training pipeline. The results from Table 4 reinforce this point; domain-specific fine-tuning on the high-quality synthetic corpus substantially elevated the performance of all tested models, suggesting that for specialized medical domains, the quality and relevance of training data is a primary determinant of model capability.

As shown in Table 5 and Fig. 6. The ablation study and hyperparameter analysis validate the efficacy of the proposed training components. The inclusion of Template-Augmented SFT confirmed the critical role of institutional templates in enforcing structural standardization. The addition of Defect-Oriented Preference Optimization yielded broad improvements, particularly in accuracy, demonstrating that learning from the model’s own errors is successful in aligning outputs more closely with expert expectations.

As shown in Table 6. The human-model comparison provides crucial insights into real-world clinical utility. The finding that UltraReporter’s reports scored higher than those of senior physicians, especially for complex and rare cases, is noteworthy. However, a subsequent clinician review revealed a key nuance: experienced physicians often prioritize efficiency, highlighting only pivotal findings and omitting information they deem non-essential. This succinct style contrasts with the comprehensive criteria used in the evaluation, which rewards detail and consequently penalized the physicians’ reports. Therefore, these results are interpreted as underscoring UltraReporter’s strength in enforcing standardization and generating consistently complete reports, rather than asserting diagnostic superiority over clinicians.

The clinician blind preference evaluation provides even more direct evidence for UltraReporter’s clinical viability. The finding that 72% of generated reports were deemed qualitatively equivalent to their physician-authored counterparts is a powerful testament to their clinical acceptability and reliability. This high tie-rate suggests that, for the majority of cases, the model’s output is indistinguishable from that of a human expert in a real-world setting.

Even more strikingly, in 12% of cases, clinicians actively preferred the model-generated reports. This may be attributed to UltraReporter’s superior consistency in adhering to institutional templates, ensuring informational completeness, and employing standardized terminology–qualities that the earlier human-model comparison also highlighted. Regarding the 16% of cases where the original reports were preferred (“Lose”), it is crucial to consider the data collection context. The spoken cues were gathered in a real-world clinical setting characterized by high patient volume and immense time pressure. To manage this workload, physicians must operate in their most efficient manner, relying on their most familiar and optimized workflow to complete diagnoses as quickly as possible. Understandably, under such pressure, they found it challenging to deviate from their ingrained habits to fully adhere to the experimental protocol of exhaustive verbalization. This resulted in an information asymmetry where the spoken cues were occasionally less comprehensive than the final reports, which were written later with direct reference to the ultrasound images. Therefore, these “lose” cases are more reflective of the inherent challenges of introducing a new protocol into a demanding clinical environment, rather than a deficiency in the model’s generative abilities.

Overall, this preference study confirms that UltraReporter not only has the potential to significantly enhance reporting efficiency but also produces reports of a quality that is explicitly endorsed by clinical professionals. It solidifies the model’s position as a valuable and reliable tool poised to assist physicians in their daily diagnostic workflow.

While the current evaluation was conducted using a standalone prototype to validate core algorithmic performance and inference latency, we have designed a comprehensive system architecture to ensure UltraReporter can be seamlessly integrated into existing hospital information ecosystems. As illustrated in Supplementary Fig.2, our proposed deployment architecture adopts a modular, tiered design consisting of three core layers to address interoperability and data security challenges:

- Clinical Environment: Embedded directly within the physician workstation UI, this layer handles audio capture and report presentation. Crucially, it integrates a Safety Mechanism. As shown in the figure, when the model’s generation confidence for critical medical entities falls below a preset threshold, the system triggers a “Low Confidence Alert,” highlighting the relevant text to enforce mandatory physician verification, thereby ensuring a robust Human-in-the-Loop workflow.
- Hospital Information Infrastructure: To ensure compatibility with existing workflows, this layer manages data interaction via a secure Gateway/API. The system utilizes HL7/FHIR protocols to communicate with the Radiology Information System for patient demographics and adopts the DICOM SR protocol to archive generated structured reports directly into the Picture Archiving and Communication System (PACS). This standards-based design eliminates the high cost of custom interface development.
- UltraReporter AI Service Layer: Acting as the backend computational engine, this layer hosts the ASR pipeline and LLM generation modules. By decoupling computation-intensive inference from the frontend clinical environment, this architecture supports deployment on local private clouds or encrypted cloud servers, maintaining the demonstrated “1-second latency” performance while adhering to hospital data privacy compliance.

This architectural blueprint demonstrates that UltraReporter is not merely an algorithmic prototype but possesses the technical feasibility for immediate implementation as a clinical-grade assistive tool.

While this study shows significant promise, certain limitations exist. The model was trained using templates from a single institution, and its adaptability to other institutional formats has not yet been tested. The training data was also in Chinese, requiring further work to validate the pipeline for other languages. We could not report ASR metrics because real-time clinical workflows precluded ground-truth transcription. Notably, the ASR system utilized pre-trained models adapted on public datasets rather than private institutional data.

## Conclusion

UltraReporter addresses the inefficiencies and inconsistencies of manual ultrasound reporting by transforming spoken diagnostic cues into structured reports using LLMs. We overcome data scarcity through Multi-Agent Data Construction, generate clinically standardized outputs via Template-Augmented SFT, and enhance diagnostic reliability with Defect-Oriented Preference Optimization. Evaluations against nine state-of-the-art LLMs demonstrate superior performance in generating accurate, thorough, and standardized reports that align with clinical workflows.

## Supplementary Information

Supplementary Information 1.

Supplementary Information 2.

## Data Availability

The datasets generated and analysed during the current study are available in the https://huggingface.co/datasets/null4785/UltraReporter.

## Code availability

The underlying code for this study can be accessed via this link https://github.com/zjl1053318729/UltraReporter.
