# Supplementary material for: UltraReporter for transforming spoken diagnostic cues into structured ultrasound reports with large language models
Source: Sci Rep. 2026 Mar 16;16:13662. doi: 10.1038/s41598-026-41439-w (PMC13125493; doi:10.1038/s41598-026-41439-w)
Supplement: Supplementary file 1 — Supplementary Information 1. [file 41598_2026_41439_MOESM1_ESM.pdf]

Checklist for Artificial Intelligence in Medical Imaging (CLAIM): 2024 Update

Section / Topic	No.	Item	Page / Line	No	NA
**TITLE / ABSTRACT**					
	**1**	Identification as a study of AI methodology, specifying the category of technology used (e.g., deep learning)	**Page 1**		
**ABSTRACT**					
	**2**	Summary of study design, methods, results, and conclusions	**Page 1**		
**INTRODUCTION**					
	**3**	Scientific and/or clinical background, including the intended use and role of the AI approach	**Page 1**		
	**4**	Study aims, objectives, and hypotheses	**Page 1**		
**METHODS**					
Study Design	**5**	Prospective or retrospective study	**Page 4** (Section 3.1 & 3.2)		
	**6**	Study goal	**Page 1** (Introduction); **Page 3** Section 3)		
Data	**7**	Data sources	**Page 3-4** (Section 3.1 & 3.2)		
	**8**	Inclusion and exclusion criteria	**Page 3-4** (Section 3.1)		
	**9**	Data pre-processing	**Page 4-5** (Section 3.2)		
	**10**	Selection of data subsets	**Page 4** (Section 3.1)		
	**11**	De-identification methods	**Page 4** (Section 3.2)		
	**12**	How missing data were handled			☑
	**13**	Image acquisition protocol			☑
Reference Standard	**14**	Definition of method(s) used to obtain reference standard	**Page 4** (Section 3.1)		
	**15**	Rationale for choosing the reference standard	**Page 4**		

			(Section 3.1)		
	**16**	Source of reference standard annotations	**Page 4** (Section 3.1)		
	**17**	Annotation of test set	**Page 4** (Section 3.1)		
	**18**	Measures of inter- and intra-rater variability of features described by the annotators	**Page 4** (Section 3.1)		
Data Partitions	**19**	How data were assigned to partitions	**Page 4** (Section 3.1)		
	**20**	Level at which partitions are disjoint	**Page 4** (Section 3.1)		
Testing Data	**21**	Intended sample size	**Page 4** (Section 3.1)		

Section / Topic	No.	Item	Page / Line	No	NA
Model	**22**	Detailed description of model	**Page 5-6** (Section 3.3)		
	**23**	Software libraries, frameworks, and packages	**Page 9** (Section 3.6)		
	**24**	Initialization of model parameters	**Page 4** (Section 3.2)		
Training	**25**	Details of training approach	**Page 5-6** (Section 3.3)		
	**26**	Method of selecting the final model	**Page 9** (Section 3.6)		
	**27**	Ensembling techniques			☑
Evaluation	**28**	Metrics of model performance	**Page 9** (Section 3.6)		
	**29**	Statistical measures of significance and uncertainty	**Page 9** (Section 3.6&4.1)		
	**30**	Robustness or sensitivity analysis	**Page 9-11** (Section 4.2)		
	**31**	Methods for explainability or interpretability	**Page 8** (Section 3.5)		
	**32**	Evaluation on internal data	**Page 4** (Section 3.1)		
	**33**	Testing on external data			☑
	**34**	Clinical trial registration			☑
**RESULTS**					
Data	**35**	Numbers of patients or examinations included and excluded	**Page 6** (Table 1) **Page 4** (Section 3.1)		
	**36**	Demographic and clinical characteristics of cases in each partition	**Page 6** (Table 1) **Page 4** (Section 3.1)		
Model performance	**37**	Performance metrics and measures of statistical uncertainty	**Page 9** (Table 2)		

Tejani AS, Klontzas ME, Gatti AA, et al. Checklist for Artificial Intelligence in Medical Imaging (CLAIM):2024 Update. Radiol Artif Intell 2024;6(4):e240300. https://doi.org/10.1148/ryai.240300

	**38**	Estimates of diagnostic performance and their precision	**Page 9** (Table 2 & Table 3)		
	**39**	Failure analysis of incorrect results	**Supplementary Material**		
**DISCUSSION**					
	**40**	Study limitations	**Page 14**		
	**41**	Implications for practice, including intended use and/or clinical role	**Page 14**		
**OTHER INFORMATION**					
	**42**	Provide a reference to the full study protocol or to additional technical details	**Page 3** (Section 3)		
	**43**	Statement about the availability of software, trained model, and/or data	**Page 15**		
	**44**	Sources of funding and other support; role of funders	**Page 17**		

* Indicate page and/or line number for each checklist item that is present. NA = not applicable.
