# Supplementary material for: UltraReporter for transforming spoken diagnostic cues into structured ultrasound reports with large language models
Source: Sci Rep. 2026 Mar 16;16:13662. doi: 10.1038/s41598-026-41439-w (PMC13125493; doi:10.1038/s41598-026-41439-w)
Supplement: Supplementary file 2 — Supplementary Information 2. [file 41598_2026_41439_MOESM2_ESM.pdf]

Supplementary Note 1: Qualitative Error Analysis

Example 1

To evaluate the cognitive burden imposed on physicians during the verification phase, we conducted a qualitative analysis of failure cases. The following illustrates a representative example of an **Anatomical Displacement Error**.

Input Prompts

> The right lobe of the thyroid gland shows no obvious abnormalities. Enlarged lymph nodes in level II of the right neck, measuring 2.1 x 0.9 cm. Enlarged lymph nodes at the **first hepatic hilum**, measuring 1.8 x 0.9 cm. Accessory spleen, measuring 1.5 x 1.1 cm. Calcification focus in the prostate. Bilateral kidneys, ureters, and bladder show no obvious abnormalities.</saying>

Predict(model generate)

> Thyroid gland size and morphology are within the normal range, with a smooth capsule. The parenchymal echogenicity of the gland is medium and fine, uniformly distributed, with no obvious abnormal echoes seen within. CDFI: Scattered blood flow signals are seen within, which are normal. Scans of both sides of the neck show multiple lymph node echoes. The larger one on the right is located in Zone II, measuring approximately 2.1 x 0.9 cm, with clear borders, oval shape, and clear internal structure. CDFI: Portal blood flow signals are seen. The larger one on the **left** is located in Zone II, measuring approximately 1.8 x 0.9 cm, with clear borders, oval shape, and clear internal structure. CDFI: Portal blood flow signals are seen. The liver is normal in size, with a smooth capsule, uniformly distributed parenchymal echogenicity, clear vascular network distribution within the liver, and no dilation of the portal vein. CDFI: No obvious abnormal blood flow signals are seen. The gallbladder is normal in size, with a smooth wall, and no abnormal echoes are seen within the sac. The common bile duct is not dilated. The pancreas is normal in size and morphology, with uniform internal echogenicity, and the main pancreatic duct is not dilated. The spleen is normal in size, with a smooth capsule, and uniform internal echogenicity. A spleen-like echo measuring approximately 1.5 x 1.1 cm is seen adjacent to the spleen. Both kidneys are normal in morphology and size, with smooth capsules, clear corticomedullary differentiation of the parenchymal echogenicity, and uniform distribution. The collecting systems of both kidneys are not separated, and no abnormal echoes are seen within. CDFI: No obvious abnormal blood flow signals are seen. Both ureters are not dilated, and no abnormal echoes are seen at the ureterovesical junction. The bladder is adequately filled, with a smooth wall and good sound transmission within. The prostate is normal in size, with clear periphery, a reasonably smooth capsule, and reasonably uniform internal echogenicity. A hyperechoic spot is seen within, accompanied by a faint shadow.</finding>No obvious abnormalities in the thyroid gland. Enlarged lymph nodes in the right side of the neck and at the first hepatic hilum. Accessory spleen. Calcification in the prostate.</impression>

Label(human write)

> The right lobe of the thyroid gland is normal in size. The capsule is smooth, the glandular tissue is evenly distributed, and no obvious abnormal mass-like echoes are detected. CDFI: Scattered blood

> flow signals are displayed internally. Scanning of both sides of the neck reveals multiple lymph node echoes. The larger ones on the right are all located in Zone II, measuring approximately 2.1cm x 0.9cm, with clear borders, oval in shape, and clear internal structure. CDFI: Gate-type blood flow signals are displayed. The liver is normal in size, with a smooth capsule, evenly distributed parenchymal echoes, clear vascular network distribution, and no dilation of the portal vein diameter. CDFI: No obvious abnormal blood flow signals are detected. Multiple lymph nodes are seen at the **first hepatic hilum**, the larger ones measuring approximately 1.8cm x 0.9cm, with clear borders, oval in shape, and clear internal structure. CDFI: Gate-type blood flow signals are displayed. The gallbladder is normal in size, with a smooth wall and no abnormal echoes within the sac. The common bile duct is not dilated. The pancreas is normal in size and shape, with uniform internal echoes and no dilation of the main pancreatic duct. The spleen is normal in size, with a smooth capsule and uniform internal echoes. A spleen-like echo measuring approximately 1.5cm x 1.1cm is seen adjacent to the spleen. Both kidneys are normal in shape and size, with smooth capsules and clear corticomedullary differentiation of parenchymal echoes, which are evenly distributed. The collecting system is not dilated, and no abnormal echoes are displayed internally. Both ureters are not dilated, and no abnormalities are seen at the ureterovesical junction. The bladder is well-filled, with a smooth wall and good sound transmission internally. The prostate is normal in size, with clear peripheral margins and a reasonably smooth capsule, and the internal echoes are reasonably evenly distributed. Bright echo spots are seen internally, accompanied by faint acoustic shadowing.</finding>No obvious abnormality in the right lobe of the thyroid. Enlarged lymph nodes in the right neck. Enlarged lymph nodes at the first hepatic hilum. Accessory spleen. Prostate calcification.</impression>

In this case, the model correctly extracted the quantitative measurements but failed to maintain the correct anatomical context in the structured narrative. However, the analysis shows that such errors often result in **internal inconsistencies** or obvious deviations from the scanning procedure, making them highly conspicuous and easily rectifiable by the reviewing physician.

Error Diagnosis & Cognitive Load Assessment:

1. **Error Type: Contextual Carryover / Anatomical Displacement**.

The model correctly captured the dimensions (1.8×0.9 cm) from the cue “First hepatic hilar lymph node” but incorrectly associated them with the “Neck” context established in the preceding sentence, fabricating a “Left side” neck finding.

2. **Cognitive Load (Low):**

While this is a generation error, it imposes low cognitive burden for detection due to **Internal Inconsistency**:

- The **Impression** section correctly lists “First hepatic hilar lymph node,” adhering strictly to the keywords in the cue.
- The **Finding** section describes a “Left neck” node.
- **Physician’s Workflow:** Upon seeing the diagnosis of “Hepatic hilar lymph node” in the Impression, the physician will naturally check the Finding section for the corresponding description. Finding it missing (and replaced by a Left Neck description they known they did not scan), the error becomes immediately apparent. The correction involves deleting the hallucinated “Left neck” text and inserting the correct hepatic template.

Example 2

Here is a case of Information Omission, where the model successfully captures the diagnostic conclusion but fails to transcribe specific quantitative measurements from the spoken cue.

Input Prompts

> Multiple thyroid nodules, category 2. Largest 0.5 x 0.3 cm. No enlarged lymph nodes seen in the bilateral cervical or supraclavicular regions. Bilateral axillary lymph nodes are visualized, measuring 1.7 x 0.6 cm on the left and 1.5 x 0.6 cm on the right. Bilateral inguinal lymph nodes are visualized, measuring 0.6 x 0.4 cm on the left and 0.7 x 0.4 cm on the right. Post-hysterectomy, no significant abnormalities noted in the abdomen.</saying>

Predict(model generate)

> Thyroid morphology and size are within the normal range, with a smooth capsule. Several hypoechoic nodules are visible within the thyroid gland. The nodules have clear boundaries, smooth margins, and are oriented horizontally. The nodules are cystic and solid in internal structure, with no obvious punctate echogenic foci observed. There is no surrounding sonolucent halo, and the posterior acoustic enhancement of the nodules is unchanged. CDFI: No significant blood flow signal is observed within the nodules. The echogenicity of the remaining glandular parenchyma is uniformly distributed. CDFI: Normal blood flow signal within the gland.
>
> No enlarged lymph nodes are observed in the bilateral cervical and supraclavicular regions.
>
> Lymph nodes are visualized in both axillary regions. The largest nodes on the left and right sides are located medially, measuring approximately 1.7*0.6cm and 1.5*0.6cm respectively. They have clear borders and clear internal structures. CDFI: demonstrates hilar blood flow signals.
>
> Lymph nodes are visualized in both inguinal regions. The largest node on the left measures approximately 0.6*0.4cm, and the largest node on the right measures approximately 0.7*0.4cm. They have clear borders and clear internal structures. CDFI: demonstrates hilar blood flow signals.
>
> The liver is normal in size, with a smooth capsule and uniform parenchymal echogenicity. The intrahepatic vascular network is clearly distributed, and the portal vein diameter is not dilated. CDFI: No significant abnormal blood flow signals are observed. The gallbladder is normal in size, with a smooth wall and no abnormal echoes within the lumen. The common bile duct is not dilated. The pancreas is normal in size and shape, with uniform internal echogenicity. The main pancreatic duct is not dilated. The spleen is normal in size, with a smooth capsule and uniform internal echogenicity.
>
> No abnormal mass-like echoes are observed in the retroperitoneum, peripancreatic region, or around the great vessels.
>
> The uterus has been removed, and no obvious abnormal echoes are seen at the stump. No significant abnormal echoes are observed in the bilateral adnexal regions.</finding> Multiple thyroid nodules (C-TIRADS Category 2) Bilateral axillary and inguinal lymph node visualization Post-hysterectomy</impression>

Label(human write)

> Thyroid size and morphology are within the normal range, with a smooth capsule. Multiple anechoic nodules are seen in the left and right lobes of the thyroid. The largest measures approximately 0.5*0.3cm, with clear boundaries, smooth edges, and a horizontal orientation. Crystalline echogenic foci are seen within the nodules, accompanied by a comet-tail artifact, without a hypoechoic halo. Posterior acoustic enhancement is noted. CDFI: No significant vascular signals are seen within the nodules. The remaining glandular parenchyma shows medium, fine, and uniformly distributed echoes. CDFI: Normal blood flow signals within the gland. No significantly enlarged lymph node echoes are seen in bilateral cervical and supraclavicular scans. Lymph nodes are visualized in bilateral axillary scans. The largest on the left measures approximately 1.70.6cm, and the largest on the right measures approximately 1.50.6cm. They are predominantly oval-shaped with clear borders and distinct internal structures. CDFI: Hilar blood flow signals are seen. Multiple lymph node-like echoes are visualized in bilateral inguinal scans. The largest on the left and right measure approximately 0.60.4cm and 0.70.4cm respectively. They have clear borders, are oval-shaped, and have distinct internal structures. CDFI: Hilar blood flow signals are seen. Liver size is normal, with a smooth capsule and uniformly distributed parenchymal echoes. The intrahepatic vascular network is clearly distributed, and the portal vein diameter is not dilated. CDFI: No significant abnormal blood flow signals are seen. Gallbladder size is normal, with a smooth wall and no abnormal echoes within the lumen. The common bile duct is not dilated. Pancreas size and morphology are normal, with uniform internal echoes and no dilation of the main pancreatic duct. Spleen size is normal, with a smooth capsule and uniform internal echoes. No abnormal mass-like echoes are seen in the retroperitoneum, peripancreatic region, and around the great vessels. Post-hysterectomy, no significant abnormal echoes are seen in the uterine region. No significant abnormal echoes are seen in the bilateral adnexal regions.</finding> Multiple thyroid nodules (C-TIRADS category 2) Bilateral axillary and inguinal lymph node visualization Status post hysterectomy</impression>

These errors represent a "conservative" failure mode. Unlike hallucinations which introduce false data, omissions result in an incomplete report. The cognitive burden here is characterized by **Recall and Insertion**: the physician notices the missing critical data (e.g., nodule size) and simply fills it in. This is a standard part of the drafting process and carries significantly lower clinical risk than unnoticed fabrications.

Error Diagnosis & Cognitive Load Assessment:

1. **Error Type: Quantitative Omission / Information Drop**.

The model correctly identified the pathology ("Thyroid nodules") and the classification ("Category 2") for the **Impression** section. However, it failed to slot the specific measurement (0.5×0.3 cm) into the generated template in the **Finding** section, likely due to the brevity of the spoken cue ("Larger 0.5 by 0.3") lacking a strong syntactic subject-verb link.

2. **Cognitive Load (Low):**

This type of error imposes a low cognitive burden due to **Template Expectation**:

- **Visual Trigger:** A standard thyroid nodule report template strictly requires dimensions (e.g., "size approx x mm"). The absence of these numbers creates a visual "gap" or a generic sentence structure that stands out to the physician.
- **Safety Profile:** The model is "fail-safe" in this scenario. It did not hallucinate a random number (e.g., "size 2.0 x 1.0"). The physician simply needs to insert the missing values dictated moments

ago. This task is an **addition** rather than a **correction**, avoiding the risk of “anchoring bias” where a physician might overlook a subtly incorrect number.

Supplementary Note 2: Figures

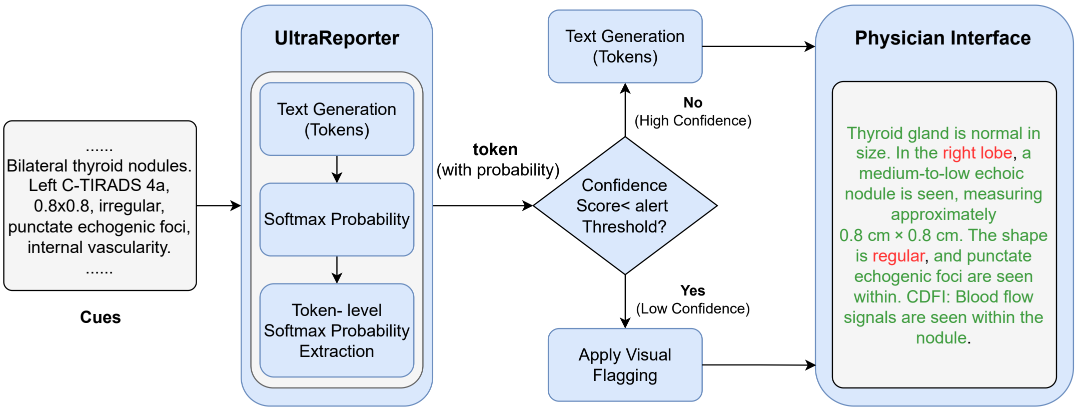

Figure 1: **Illustration of the Confidence-Based Safety Mechanism.** To prevent automation bias and ensure clinical safety, UltraReporter incorporates a real-time uncertainty monitoring module. The system extracts token-level softmax probabilities during text generation. If the confidence score for a critical medical entity (e.g., measurements, descriptors) falls below a pre-set alert threshold, the specific text segment is automatically flagged (e.g., highlighted in red) on the Physician Interface. This "Human-in-the-Loop" design directs the physician's attention to low-confidence areas for mandatory verification before the report is finalized.

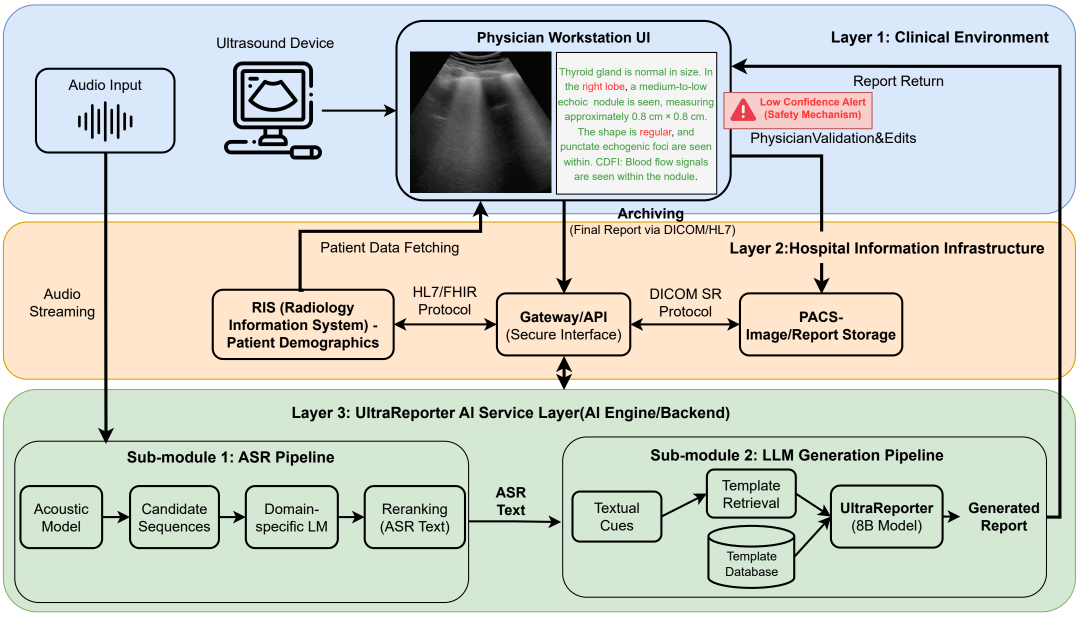

Figure 2: **Proposed Clinical Deployment Architecture.** The system is designed with a three-layer modular architecture to ensure seamless integration into existing hospital workflows: **Layer 1 (Clinical Environment):** Captures audio at the point of care and presents the generated report with integrated safety alerts for physician validation. **Layer 2 (Hospital Information Infrastructure):** Manages secure data interoperability, utilizing HL7/FHIR protocols to fetch patient demographics from the RIS and DICOM SR protocols to archive final reports into the PACS. **Layer 3 (AI Service Layer):** Decouples heavy computation (ASR and LLM pipelines) from the frontend, enabling the "one-second latency" performance via a dedicated backend engine while ensuring data security.
